# Role of carnoy’s solution in the treatment of keratocystic odontogenic tumor: A systematic review

**DOI:** 10.4317/medoral.21250

**Published:** 2016-07-31

**Authors:** Álvaro Díaz-Belenguer, Alba Sánchez-Torres, Cosme Gay-Escoda

**Affiliations:** 1 DDS. Fellow of the Master’s Degree Program in Oral Surgery (EHFRE International University/FUCSO); 2 DDS. Fellow of the Master of Oral Surgery and Orofacial Implantology. School of Dentistry, University of Barcelona, Spain; 3 MD, DDS, MS, PhD, EBOS. Chairman and Professor of the Oral and Maxillofacial Surgery Department, School of Dentistry, University of Barcelona. Director of Master’s Degree Program in Oral Surgery and Implantology (EHFRE International University/FUCSO). Coordinator/Researcher of the IDIBELL Institute. Head of Oral and Maxillofacial Surgery and Implantology Department of the Teknon Medical Center, Barcelona, Spain

## Abstract

**Introduction and Objective:**

The keratocystic odontogenic tumor is a benign but aggressive neoplasm. As enucleation alone obtains high recurrence rates, some adjuvant treatments such as Carnoy’s solution have been proposed. The aim of this study is to evaluate the reduction of recurrences with the use of Carnoy’s solution as adjuvant in the treatment of keratocystic odontogenic tumors.

**Material and Methods:**

An electronic search in Pubmed (MEDLINE), ScienceDirect and Cochrane databases was conducted with the key words “odontogenic keratocyst”, “keratocystic odontogenic tumor”, “carnoy’s solution”, “treatment” and “enucleation”. The inclusion criteria were clinical studies using Carnoy’s solution as adjuvant for the treatment of keratocystic odontogenic tumors, published in English, including at least 10 patients. Articles with an unclear reporting of the treatment applied, nonhuman studies, case reports and lesions associated to Gorlin-Goltz syndrome were excluded.

**Results:**

All the studies included were case series. The recurrence rate of enucleation ranged from 0% to 58.8%. With the only use of Carnoy’s solution as adjuvant treatment to the enucleation, recurrences varied from 0% to 100%. The use of ≥ 2 adjuvant treatments reduced the range between 0% and 7.9%.

**Conclusions:**

The use of Carnoy’s solution as adjuvant therapy for the treatment of keratocystic odontogenic tumor has a grade C recommendation.

**Key words:**Carnoy’s solution, keratocystic odontogenic tumor, treatment, recurrence.

## Introduction

The odontogenic keratocyst was first described by Philipsen in 1956 (1). From the two histologic variants, the orthokeratinized one appears in 12% of cases and the parakeratinized variant of this entity in 90% of them (1,2). The later was renamed as keratocystic odontogenic tumor (KOT) by the World Health Organization (WHO) in 2005 that is a benign but aggressive neoplasm of odontogenic origin, histologically characterized by a thin parakeratinized stratified squamous epithelium (1-9). Some cell proliferation markers as Ki-7, PCNA or p53 have been found in the suprabasal zone (10). This tumor can appear as a single or multiple lesions and even, as a part of nevoid basal cell carcinoma syndrome or Gorlin-Goltz syndrome (1,4,10). The higher incidence appears in patients ranging from 20 to 40 years old and its prevalence is higher in men than in women, in a 2:1 proportion (9,11).

The histopathological analysis is necessary to establish the definitive diagnosis. An incisional or excisional biopsy, or a fine-needle aspiration biopsy are the most used techniques to obtain a sample (2), although the presence of inflammatory infiltrate can impair the diagnosis and give rise to false negatives (1,2).

Radiologically, it appears as a unilocular or multilocular well-defined lesion with scalloped margins (10). It can be associated with an included tooth. The differential diagnosis includes the dentigerous or follicular cyst, radicular cyst, periodontal lateral cyst and ameloblastoma, among others (2,9,12). The most frequent location is in mandible (70-75%), particularly in the angle and mandibular ramus (1,5,9-11,13).

High recurrence rates have been described for this lesion (4,5,9,12,14) mainly depending on the treatment modality as it will determine a complete or incomplete cyst removal (3,7). In addition, a new primary cyst formation in the proximity of the former could be interpreted as a recurrence (8).

Enucleation is the treatment of choice although it can result in a 60% of recurrences (9). Thus, other adjuvant treatments such as cryotherapy, peripheral ostectomy with rotary instruments, excision of the adhered mucosa, electrocoagulation, Carnoy’s solution, marsupialization, decompression and secondary excision or resection have been used (4,6-8,12).

Carnoy’s solution (CS) is a cauterizing agent that causes a rapid local fixation. The solution can be used inside the cyst to facilitate a complete remotion of the cystic membrane or directly over the bony bed after the cyst enucleation to detect and eliminate the remaining epithelium of the KOT to diminish the likelihood of a recurrence (5).

The aim of this systematic review was to evaluate the reduction of recurrences associated to the application of Carnoy’s solution as adjuvant in the treatment of keratocystic odontogenic tumors.

## Material and Methods

This article follows the Preferred Reporting Items for Systematic Reviews and Meta-Analyses (PRISMA) declaration (15). The selected articles were classified in different levels of evidence by means of the Strength of Recommendation Taxonomy (SORT) criteria (16).

An electronic search in Pubmed (MEDLINE), ScienceDirect and Cochrane databases. The last search was conducted on 27th February 2015. The keywords used were “odontogenic keratocyst”, “keratocystic odontogenic tumor”, “carnoy’s solution”, “treatment” and “enucleation”. The Boolean operator “AND” was used in order to obtain the most relevant studies.

The inclusion criteria were prospective or retrospective clinical studies using CS as adjuvant treatment for the treatment of primary KOT, articles published in English, including at least 10 patients with a diagnosed KOT. The exclusion criteria were studies with unclear reporting of the treatment applied, nonhuman studies, case reports and lesions associated to Gorlin-Goltz syndrome.

The articles selection was agreed by consensus between two of the authors; first by reading of titles and abstracts of the found bibliographic cites to identify the most rele-vant studies and then, by means of reading the full-text. No metaanalysis could be done due to the heterogeneity of the studies included.

## Results

**Figure 1:**
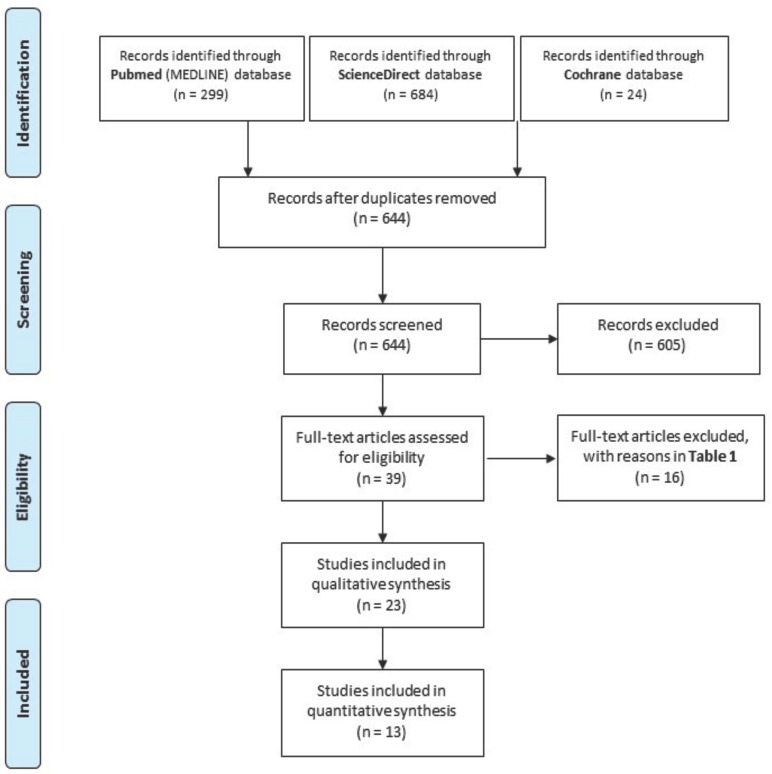
Flow of articles through the systematic review according to the PRISMA statement.

The flow chart of the selected articles can be seen in figure 1. Among the 644 studies initially obtained from the search, 605 were excluded by reading the title and abstract. Thus, the complete text of 39 articles was analyzed by the two authors. Sixteen articles (17-32) were excluded; the reasons are detailed in  Table 1. Thus, twenty-three articles with relevance were selected to be included in the systematic review: 5 systematic reviews (6,7,9,33,34), 4 reviews (1,2,10,11), 10 retrospective (3-5,8,14,35-40) and 3 prospective studies (12,13,41). Concretely, 13 articles were subjected to data extraction and complete analysis. Despite all these studies had a scientific level 3 and no randomized clinical trials could be found, the authors decided to include them to analyze the available literature. The main results of the included clinical studies are shown in  Table 2. It should be noted that the studies performed by Zhao *et al.* (14) and Rao and Kumar (37) did not clearly specify the presence of the parakeratotic component.

The recurrence rates for enucleation varied from 0 to 58.8% in a period of time ranging from 3 months to 16 years. The use of Carnoy’s solution as adjuvant to enucleation had recurrence rates from 0 to 100% in a time period of 3 months to 9 years. Interestingly, the use of two or more adjuvant techniques had a 0 to 7.9% of recurrences between 1 and 16 years. Even though, the treatments that included two or more adjuvant techniques were performed in less patients than the others.

**Table 1:**
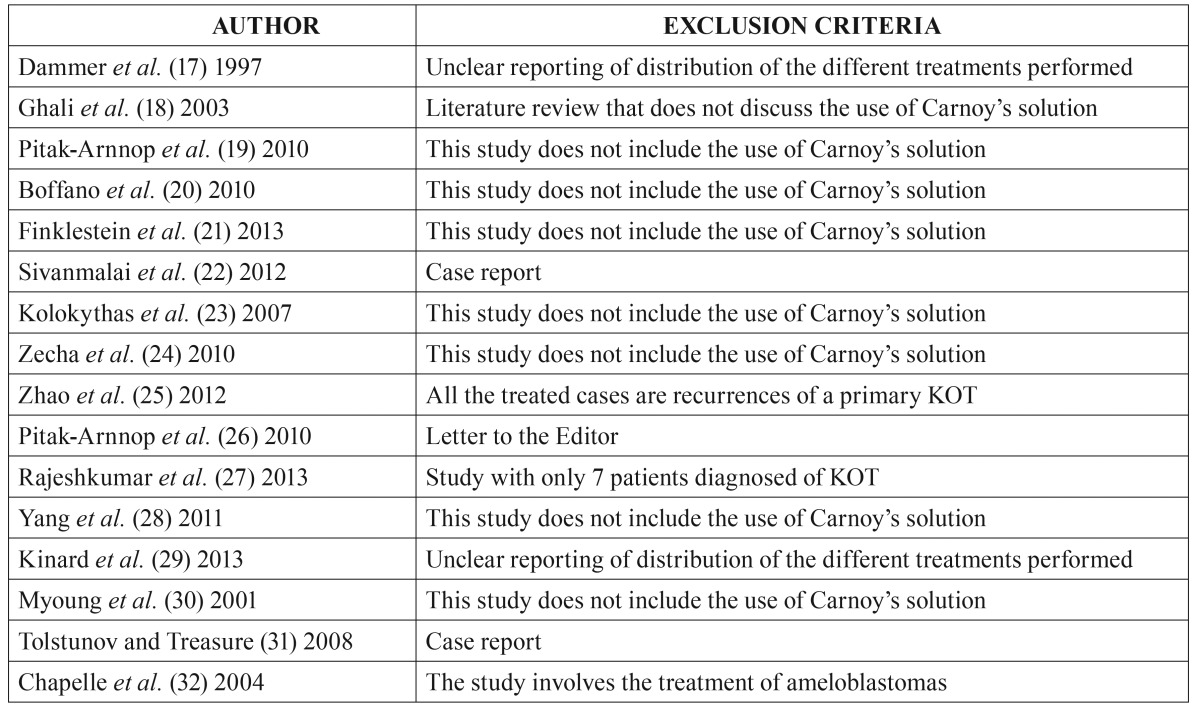
List of excluded studies and reasons.

**Table 2:**
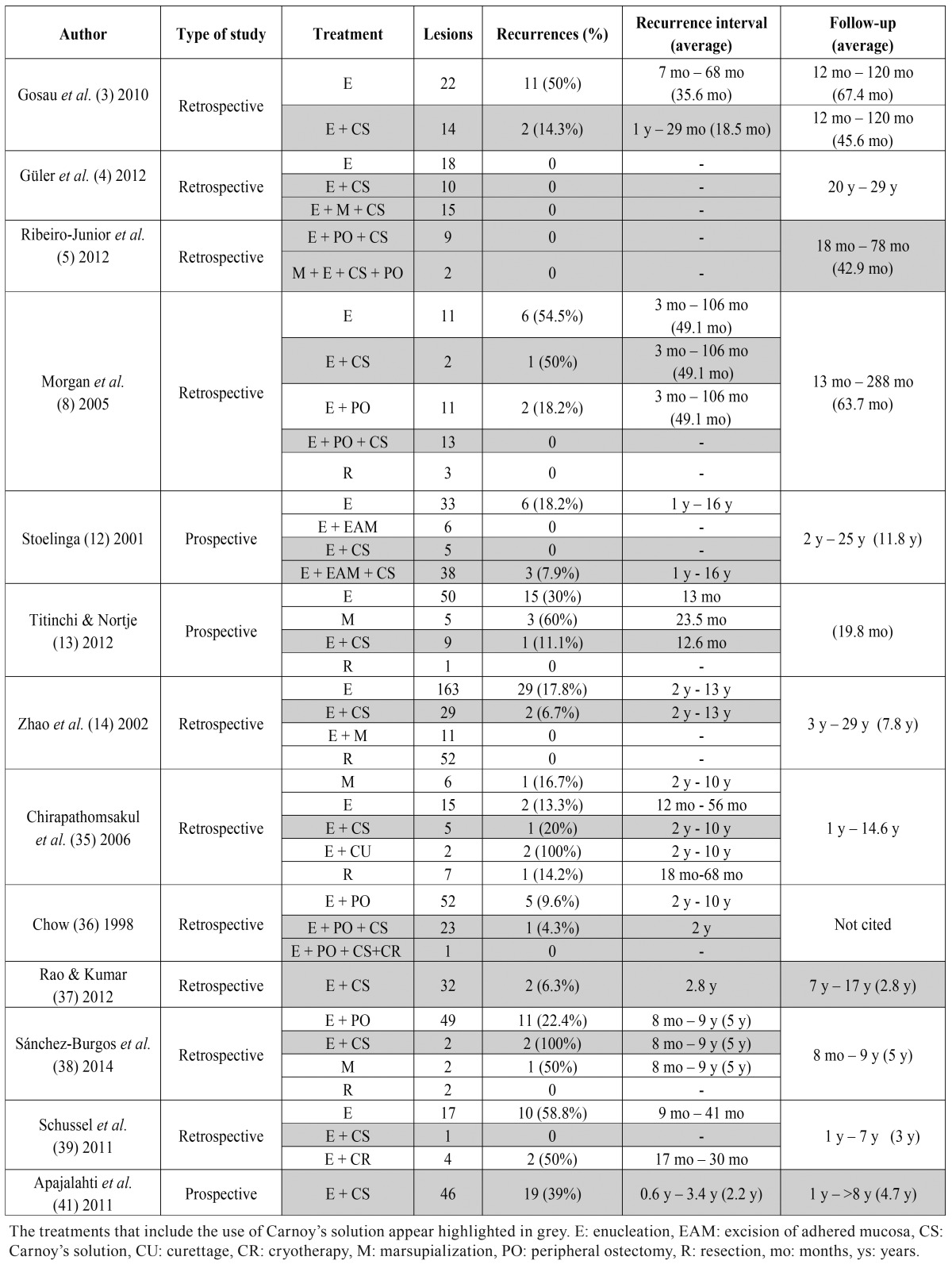
Characteristics of the clinical studies included in this systematic review regarding to the type of study, the treatments performed, the recurrences and the time of recurrence.

## Discussion

As previously explained, KOT is a benign but aggressive neoplasm of odontogenic origin (3-9). High recurrence rates have been described for this lesion (4,5,9,12,14,37) mainly depending on the treatment modality (3,7). Accordingly, a recently published study (40) quantified Cyclin D1 (CCD1) expression levels, a nuclear protein essential for cell cycle progression, in a series of keratin-producing odontogenic cysts. They concluded that these levels did not predict non syndromic KOT recurrences and that factors not related to the lesion biology could have an influence in the recurrence rates.

Regarding to radiological appearance, lesions with scalloped and often corticated margins or a multilobular and multilocular appearance can be observed (4,9,12,14,35). Displacement of impacted or erupted teeth, root resorption, root displacement or extrusion of erupted teeth can occur (4). These lesions are difficult to interpret and easy to be confused with other lesions (12,14). Often asymptomatic, the majority of KOT tends to appear at the mandible, frequently at the mandibular ramus and angle (3,4,9,14,37), although it can also occur in the dentate area of the jaws resembling an odontogenic cyst (12). Even, involvement with an impacted tooth has been described up to 40% of cases (4,35).

The different treatment modalities can be divided in conservative methods such as enucleation, decompression or marsupialization and in invasive ones, that is, cryosurgery or resection (6,36). Although the most radical treatments have shown the lower recurrence rates, available evidence does not demonstrate the most effective technique in terms of morbidity and recurrence prevention (3,5,6,8,38).

Enucleation is the most commonly used method to treat the majority of KOT, although with a high rate of recurrences (9). Thus, adjuvant techniques such as the use of CS either before the cyst enucleation or placed directly in the bony bed after the enucleation have been proposed to eliminate residual tissue and so prevent recurrences (3,5,7,9,14). Although CS was initially described to be placed into the cyst lumen before enucleation, most clinicians apply it after (7,33). This aspect could introduce a bias at the time of analyzing the recurrence rates. CS is a cauterizing and fixating agent that penetrates cancellous spaces in the bone (3,6,36). The time of application is sufficient for 10 to 15 minutes, although if inferior alveolar nerve is visible into the cyst cavity, the application cannot last for more than 3 minutes because of damage of nerve fibers has been described (14).

Some authors defend that techniques such as marsupialization with posterior enucleation are better for larger cysts to reduce the morbidity and to be more conservative (7,9,14). Others state that more invasive techniques such as resection have to be reserved for recurrent KOT with the aim to eliminate satellite cysts or epithelial remnants (3,7,14).

The different adjuvant techniques used in the studies included in this systematic review for the treatment of KOT difficult the analysis of the application of CS as a unique adjuvant treatment apart from the enucleation. CS has been used in combination with enucleation, peripheral ostectomy, curettage, marsupialization and excision of affected mucosa. As found in this study, not only the use of CS but also the use of multiple adjuvant treatments reduce the recurrence rates compared to enucleation alone.

Regarding to the limitations of the included studies, the differences in the number of participants around the distinct treatments performed difficult to draw conclusions. In a study made by Chow (36), one patient was treated by means of enucleation, CS, peripheral ostectomy and cryotherapy. Although the lesion did not recurred it is not possible to state that the combination of this techniques yields the best results, similarly to other treatments performed in some of the included studies (5,8,12,35). The studies published by Stoelinga (12), Zhao *et al.* (14) (both published prior to 2005) and Güler *et al.* (4) and Rao and Kumar (37) did not specify the histologic variant of the treated odontogenic keratocysts. Thus, a risk of bias in the results could have been introduced. In the retrospective study of Ribeiro *et al.* (5), they did not specify in which cases the excision of mucosa was done. With regard to the cases that had been followed up, the study from Guler *et al.* (4) only revisited a 32.5% of patients and Rao and Kumar (37) did only control visits in 12 from 32 cases.

The retrospective nature of some of the included studies has several limitations such as different lengths of follow-up. The study of Gosau *et al.* (3) found that lesions treated by means of enucleation plus CS had a recurrence rate of 14.3% whereas the cases treated with enucleation had a 50% of recurrences. However, the length of follow-up was shorter in the first group (18.5 months as average) than in the enucleation group (36.5 months). Most studies recommend the need of a long-term follow-up at regular intervals after surgery (7,8,35,38,39). A follow-up of 5 years or more is recommended because some recurrences have been reported 16 years after the initial treatment (12). Morgan *et al.* (8) studied the possibility of having more recurrences because of a longer follow-up and they did not found significant differences between the mean time to recurrence for patients with recurrence and the mean follow-up for patients without recurrence. As stated by Antonoglou *et al.* (34), the different times of follow-up among the studies and the lack of histopathologic data in some of them could introduce a bias in the results. Moreover, there are no randomized clinical trials performed for the treatment of KOT, as stated by the Sharif and Oliver systematic review (9).

Future research with randomized and/or controlled clinical studies, with similar samples or number of lesions treated and with a long-term follow-up is needed to obtain consistent results.

## Conclusions

The lack of randomized clinical trials, the methodological differences and the low level of evidence of the included studies allow to conclude that the use of Carnoy’s solution as adjuvant therapy for the treatment of KOT has a grade C recommendation and that there is not a clear reduction in the recurrences.
